# Genotoxicity of micro- and nano-particles of kaolin in human primary dermal keratinocytes and fibroblasts

**DOI:** 10.1186/s41021-020-00155-1

**Published:** 2020-04-16

**Authors:** Masanobu Kawanishi, Reimi Yoneda, Yukari Totsuka, Takashi Yagi

**Affiliations:** 1 Graduate School of Science and Radiation Research Center, Osaka Prefecture University, 1-2 Gakuen-cho, Nakaku, Sakai-city, Osaka, 599-8570 Japan; 2 Division of Carcinogenesis and Prevention, National Cancer Center Research Institute, Tokyo, Japan

**Keywords:** Genotoxicity, Kaolin, Nanoparticle, Primary human keratinocyte

## Abstract

**Introduction:**

Kaolin is a clay mineral with the chemical composition Al_2_Si_2_O_5_(OH)_4_. It is an important industrial material, and is also used as a white cosmetic pigment. We previously reported that fine particles of kaolin have genotoxic potency to Chinese hamster ovary CHO AA8 cells, and to the lungs of C57BL/6 J and ICR mice. In the present study, we evaluated the genotoxicity of different particle sizes of kaolin using primary normal human diploid epidermal keratinocytes and primary normal human diploid dermal fibroblasts, in addition to a CHO AA8 cell line.

**Findings:**

After 6-h treatment with kaolin micro- and nano-particles of particle sizes 4.8 μm and 0.2 μm (200 nm), respectively, the frequencies of micronucleated cells increased in a dose-dependent manner. The frequency increased 3- to 4-fold by exposure to the particles at 200 μg/mL (i.e.*,* 31.4 μg/cm^2^) in all cells tested. Two-way ANOVA revealed a significant main effect of particle size, and the nano-particles tended to have a higher potency of micronucleus (MN) induction. However, the cell type did not significantly affect the MN frequencies. In addition, one-hour treatment with the kaolin particles increased DNA damage in a dose-dependent manner in a comet assay. The %tail DNA was increased 8- to 20-fold by exposure to the particles at 200 μg/mL, for all cells tested. The kaolin nano-particles had higher DNA-damaging potency than the micro-particles. Furthermore, treatment with kaolin particles dose-dependently increased the production of reactive oxygen species (ROS) in all cells. Again, we observed that kaolin nano-particles induced more ROS than the micro-particles in all cells.

**Conclusion:**

Kaolin particles demonstrated genotoxicity in primary normal human diploid epidermal keratinocytes and fibroblasts as well as in CHO AA8 cells. Although no significant difference was observed among these three types of cells, fine particles of kaolin tended to have higher genotoxic potency than coarse particles. Since studies on its genotoxicity to skin have been scarce, the findings of the present study could contribute to safety evaluations of kaolin particles when used as a white cosmetic pigment.

## Introduction

Kaolin is a clay mineral with the chemical composition Al_2_Si_2_O_5_(OH)_4_. It is used in large quantities by numerous industries including paper production, paint, rubber, plastics, ceramics, chemicals, pharmaceuticals, and cosmetics [1]. We have previously reported that kaolin particles showed genotoxic effects in in vitro and in vivo assay systems [2–4]. For example, kaolin nano-particles induce micronuclei in both Chinese hamster ovary (CHO) AA8 and human lung cancer A549 cell lines in micronucleus (MN) test, and DNA damages in lung of C57BL/6 J mice in comet assay [3, 4].

Because of their useful physical and chemical characteristics such as increased chemical reactivity, larger active surface area, and enhanced electrical conductivity, nanosized particles are important materials in many areas including the industrial, medical, and cosmetic fields [5, 6]. However, their adverse effects on health have begun to be reported in recent years, and there have been many reports indicating that toxicity induced by fine particles is influenced by physicochemical differences such as size [5, 6]. To date, however, toxicological data on the effects of nanoparticles are not fully consistent [6].

The species of laboratory animals and types of cells used in toxicity tests are known to affect the results. Although toxicological data for kaolin through inhalation have accumulated, since it is an important industrial mineral, those for transdermal genotoxicity are also important because it is used as a white cosmetic pigment. Furthermore, Ben-David et al. recently reported that cell lines used in laboratories acquire genetic and transcriptional heterogeneity during culture, resulting in alterations of the drug response [7]. Therefore, in the present study, the genotoxicities of different particle sizes of kaolin (micro- and nano-particles) were evaluated using primary normal human diploid epidermal keratinocytes and primary normal human diploid dermal fibroblasts, as well as a CHO cell line. We assessed the genotoxicity with the in vitro MN test and the induction of DNA damage with the comet assay. We also measured reactive oxygen species (ROS) production in cells treated with the particles.

## Materials and methods

### Kaolin particles and cells

Kaolin micro-particles and nano-particles with median sedigraph particle sizes of 4.8 μm and 0.2 μm (200 nm), respectively, were purchased from Engelhard Corporation, (Iselin, NJ, USA). Crystal appearance observed under a scanning electron microscope (SEM) was done by AKIT Corporation (Gifu, Japan). The size distributions of the particles in the SEM images were analyzed with the assistance of ImageJ FIJI software (National Institutes of Health, Bethesda, MD, USA). The particles were suspended in saline (Otsuka Pharmaceutical, Tokyo, Japan) containing 0.05% of Tween 80 (Nacalai Tesque, Kyoto, Japan). Primary normal human epidermal keratinocytes, neonatal (HEKn) were obtained from Gibco Thermo Fisher Scientific (Tokyo, Japan). Primary normal human dermal fibroblast cells FJ were prepared as described previously [8]. CHO cell line AA8 was obtained from RIKEN BioResource Research Center (Ibaragi, Japan).

### Micronucleus test

The MN test was carried out as previously described [9]. Briefly, FJ cells and CHO AA8 cells were cultured in RPMI-1640 medium (Sigma-Aldrich Japan, Tokyo, Japan) supplemented with 10% fetal bovine serum (JRH Biosciences, Lenexa, KS, USA) at 37 °C in a 5% CO_2_ atmosphere. HEKn was cultured in HuMEDIA-KG2 (Gibco). The cells (HEKn and FJ, 7 × 10^5^ cells/dish; CHO AA8, 4 × 10^5^ cells/dish) were then seeded in plastic cell culture dishes (ϕ60 mm) one day before the treatment procedure. Suspensions of the nanoparticles were sonicated for 5–10 min at room temperature, and one volume of the suspension was mixed with 9 volumes of the culture medium supplemented with 10% fetal bovine serum (total: 3.3 mL/dish). The cells were then treated with the particles at the indicated concentrations for 6 h. As a positive control, mitomycin C (MMC; final concentration 0.1 μg/mL) was used. After being treated, the human and Chinese hamster cells were cultured for a further 42 or 20 h, respectively. The cells were then trypsinized, counted, and centrifuged. Growth inhibition was calculated using the following formula:

Relative cell growth = (number of treated cells) ÷ (number of non-treated cells).

The cells were then resuspended in 0.075 M KCl and fixed in methanol:glacial acetic acid (3:1) and washed with methanol containing 1% acetic acid. Finally, the cells were resuspended in methanol containing 1% acetic acid. The nuclei were stained with acridine orange (Nacalai Tesque) and observed using fluorescence microscopy. The number of cells with MN was recorded based on the observation of 1000 interphase cells in each experiment.

### Comet assay

The cells were treated with kaolin particles as described in the previous section. After 1 h of treatment, the cells were collected and DNA strand breaks were quantified with single cell alkaline agarose gel electrophoresis using a CometAssay kit (Cosmo Bio, Tokyo, Japan). Electrophoresis was performed in the alkaline buffer at 21 V 300 mA for 30 min. The gel was stained with SYBR Green I solution (Cosmo Bio). The tail length was calculated from 40 to 100 comet images with CASPLab softwere (CASPLab.com).

### ROS detection

The cells were treated with kaolin particles as described in the previous section. After 1 h of treatment, the cells were collected and stained for 15 min with 10 μM H_2_DCFDA (dichlorodihydrofluorescein diacetate, Life Technologies, Thermo Fisher Scientific, Tokyo, Japan). The fluorescent signals induced by cellular ROS were recorded with a Tali image-based cytometer (Life Technologies, Thermo Fisher Scientific). Frequencies of ROS positive cells were measured, and then, relative frequencies to those in control treatments were calculated.

### Statistical analysis

Two-way or one-way analysis of variance (ANOVA) was carried out when appropriate. Student’s *t*-test with the Bonferroni correction or Tukey’s test was used for pair-wise comparisons.

## Results and discussion

### Appearance of particles and induction of micronucleus

**Fig. 1:**
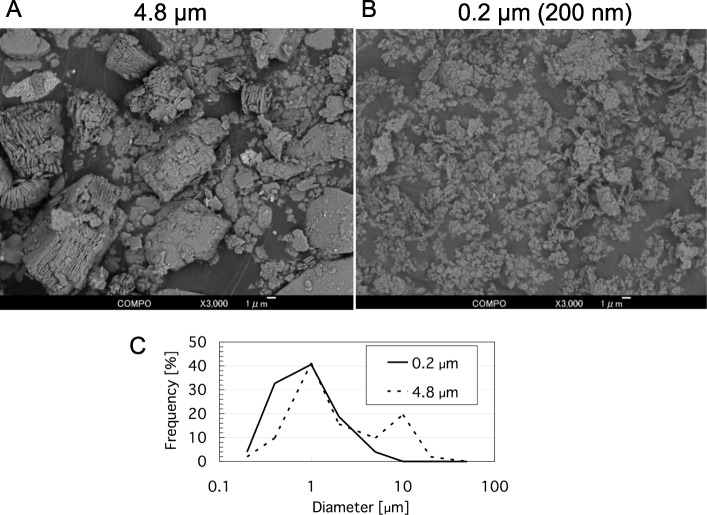
SEM images of kaolin micro- and nano-particles. The reflected electron images of micro-particles (**a**; 4.8 μm) and nano-particles (**b**; 0.2 μm) were obtained at E = 5 kV, × 3000. Scale bar indicates 1 μm. **c**: The size distributions of the particles

**Fig. 2:**
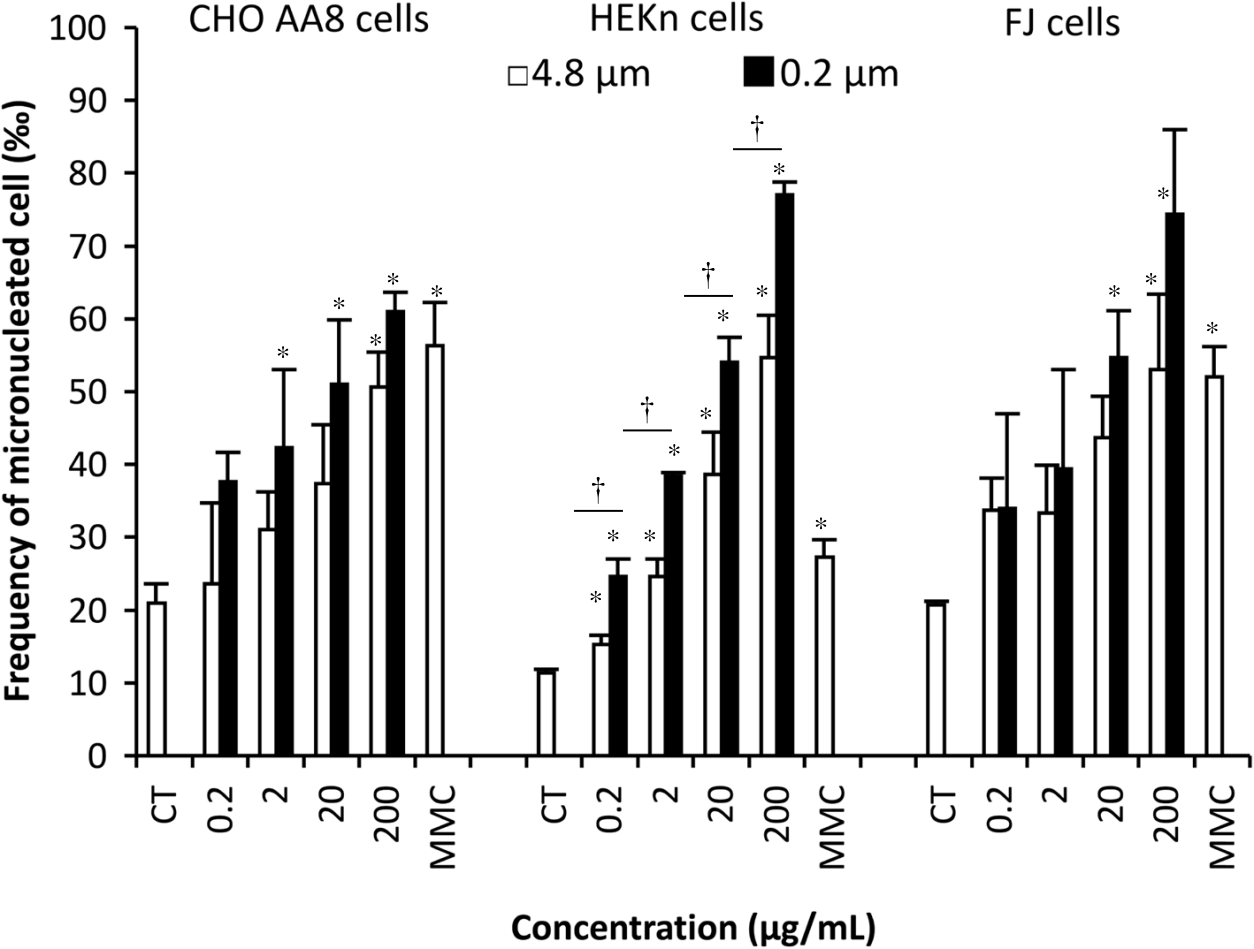
Micronuclei formation in Chinese hamster ovary CHO AA8, primary normal human epidermal keratinocytes neonatal (HEKn) and primary normal human dermal fibroblast FJ cells after 6 h’ treatment with kaolin micro- and nano-particles. Mean ± S.D. values of three independent experiments are shown. In the graph, CT represents the solvent control (treatment with 0.005% (v/v) Tween 80). Concentrations are given in μg/mL. MMC represents a positive control (0.1 μg/mL mitomycin C) treatment. *Significant difference versus the solvent control at *p* < 0.05, †significant difference between particle size at *p* < 0.05, according to the Tukey-Kramer method

Figure 1 shows SEM images of the kaolin micro- and nano-particles (4.8 μm and 200 nm, respectively). The micro-particles were coarse, while nano-particles were fine. Kaolin micro-particles showed a bimodal distribution with one peak at 1 μm and the other at 10 μm, and particles smaller than 0.4 μm were 10%. On the other hand, the distribution of nano-particles did not have the peak at 10 μm, and 35% of the particles were smaller than 0.4 μm (Fig. 1c). The MN-inducing activities of the kaolin micro- and nano-particles were examined using the Chinese hamster ovary cell line CHO AA8, which is commonly used in the test, primary normal human diploid epidermal keratinocyte neonatal HEKn cells, and primary normal human diploid dermal fibroblast cells FJ. By the treatment with 200 μg/mL (31.4 μg/cm^2^) kaolin micro- and nano-particles, the relative growths of CHO AA8 cells reduced to 68 and 53%, respectively. Those of the HEKn cells were 75 and 68%, and those of FJ cells were 83 and 74%, respectively. As shown in Fig. 2, the kaolin micro-particles increased the number of MN cells in a dose-dependent manner. The background MN frequencies of CHO AA8, HEKn and FJ cells were 21‰, 11‰ and 21‰, respectively. With kaolin micro-particle treatment at a concentration of 200 μg/mL, the frequencies of CHO AA8, HEKn and FJ cells rose to 51‰, 55‰, and 53‰, respectively. Using kaolin nano-particles at 200 μg/mL, the frequencies of CHO AA8, HEKn and FJ cells rose to 61‰, 77‰ and 74‰, respectively. Two-way ANOVA revealed that the main effects of concentration and particle size on the MN-induction were significant (both *p* < 0.05) in all cells, and in HEKn cells their interaction effect was also significant (*p* < 0.05). However, the effect of cell type on the MN-induction was not significant (*p* > 0.05). The Tukey test showed that the increases of MN frequencies compared with solvent controls were significant (*p* < 0.05) at concentrations ≥2, ≥0.2 and ≥ 20 μg/mL in CHO AA8, HEKn and FJ cells, respectively. According to the Tukey test, nano-particles induced significantly (*p* < 0.05) more MN than micro-particles in HEKn cells at all concentrations tested. Pair-wise comparisons (Student’s *t*-test without Bonferroni correction) showed significant differences (*p* < 0.05) between nano- and micro-particles for the treatments at ≥20 μg/mL (FJ) and 200 μg/mL (CHO AA8), but the differences were not significant in the Tukey test. Although we cannot exclude the contribution of fine particles in the MN-induction by the micro-particles since the micro-particles contained 10% of grains smaller than 0.4 μm as shown in Fig. 1c, these data indicate that both micro- and nano-particles of kaolin are genotoxic to all three cell types. Fine particles tend to have higher genotoxicity than coarse ones (though statistically significant only for HEKn cells), and the cell type does not affect the MN-induction among these three types of cell.

### DNA damage induction

**Fig. 3:**
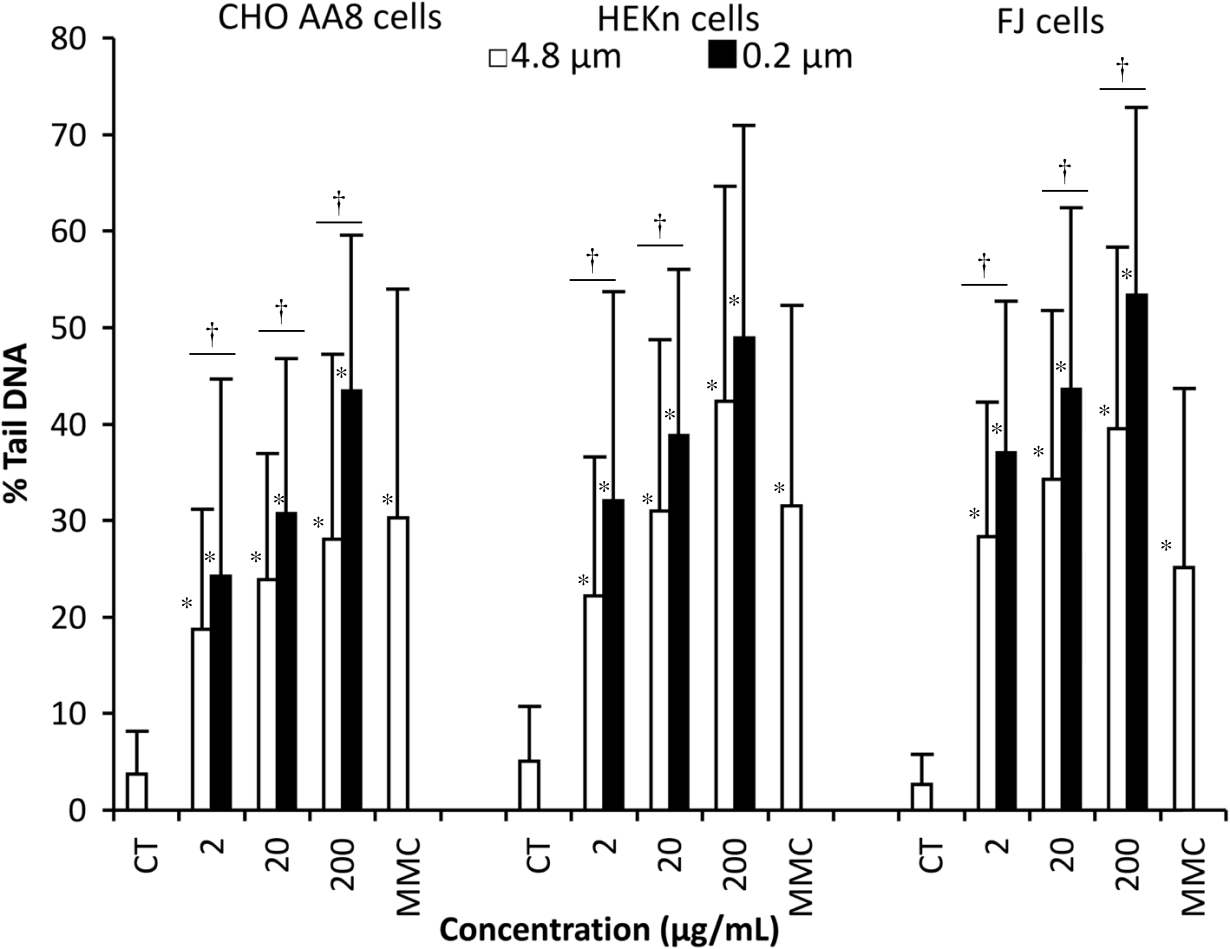
Induction of DNA damage in CHO AA8, HEKn and FJ cells by 1 h’s treatment with kaolin micro- and nano-particles. Mean ± S.D. values of 40–100 comet images are shown. In the graph, CT represents the solvent control (treatment with 0.005% (v/v) Tween 80). Concentrations are given in μg/mL. MMC represents 0.1 μg/mL MMC-treatment as a reference. *Significant difference versus the solvent control at *p* < 0.0167, †significant difference between particle size at *p* < 0.0167, according to the *t*-test with Bonferroni correction

Next, we measured DNA damage in the cells treated with the kaolin particles using the comet assay, since MN can be induced by DNA damage. As shown in Fig. 3, one-hour treatment with the kaolin particles increased the comet tail length in a dose-dependent manner. The background %tail DNA values for CHO AA8, HEKn and FJ cells were 3.7, 5.1 and 2.7%, respectively. After the treatment with kaolin micro-particles at 200 μg/mL, the values for CHO AA8, HEKn and FJ cells rose to 28, 42 and 40%, respectively. At a kaolin nano-particle concentration of 200 μg/mL, the %tail DNA values of CHO AA8, HEKn and FJ cells were 43, 49 and 53%, respectively. Two-way ANOVA revealed significant main effects of concentration and particle size on DNA-damage induction (both *p* < 0.05) in all cells, and their interaction effect was also significant (*p* < 0.05) in all cells. Student’s *t*-test with the Bonferroni correction showed that the increases of DNA damage compared with the solvent control treatment were significant (*p* < 0.0167) in all three cells at all concentrations tested. According to the *t*-test with the Bonferroni correction, nano-particles induced significantly (*p* < 0.0167) more DNA damage than micro-particles in CHO AA8 and FJ cells at all concentrations tested, whereas in HEKn cells significance (*p* < 0.0167) was observed at 2 and 20 μg/mL. We concluded that both micro- and nano-particles induced DNA damage in all three cells, and fine particles had higher DNA-damaging potency than coarse particles.

### ROS production

**Fig. 4:**
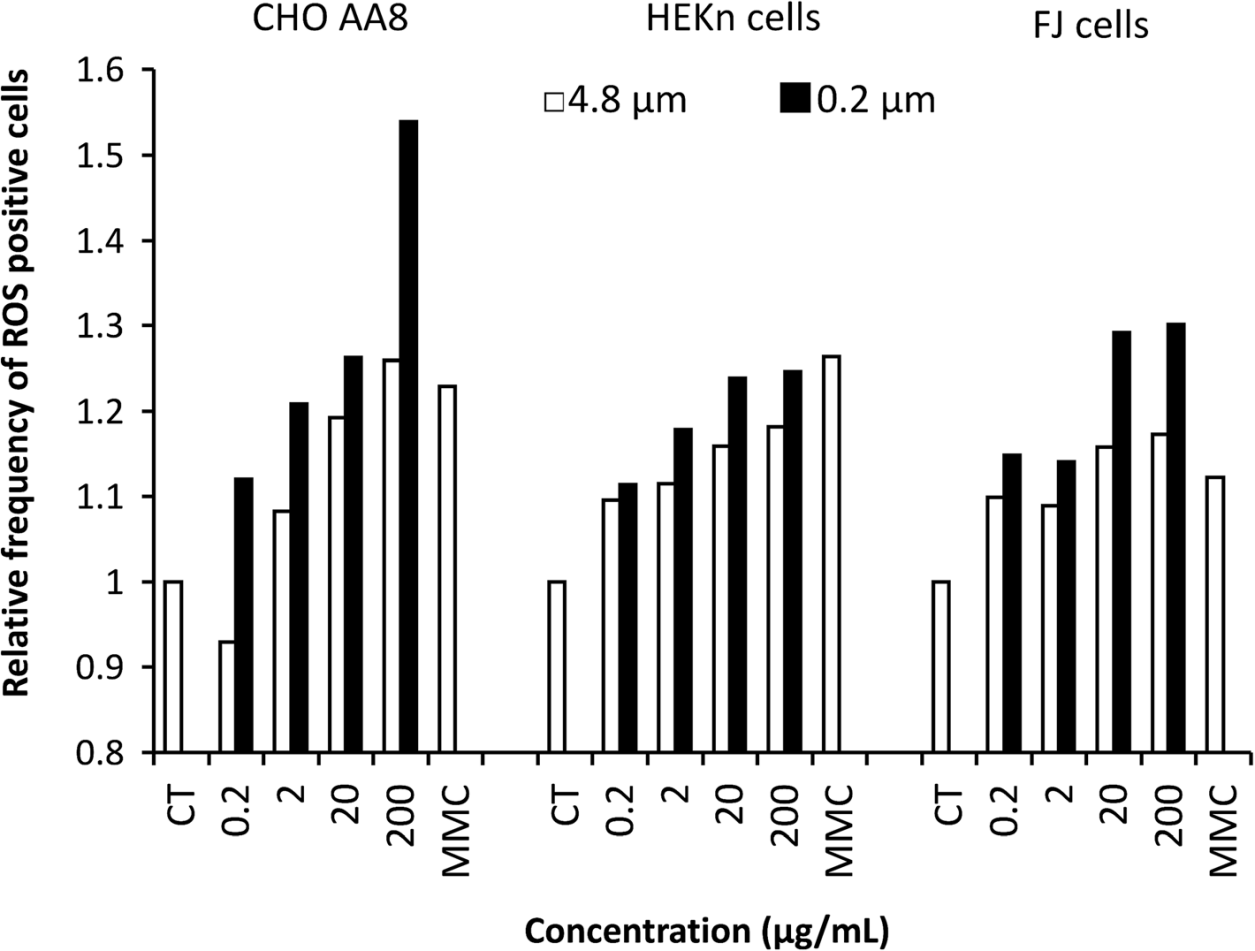
Production of ROS in CHO AA8, HEKn and FJ cells by 1 h’s treatment with kaolin micro- and nano-particles. Relative frequencies of ROS positive cells (mean values) to that of the solvent control treatment are shown. In the graph, CT represents the solvent control (treatment with 0.005% (v/v) Tween 80). Concentrations are given in μg/mL. MMC represents 0.1 μg/mL MMC treatment

One of the major mechanisms by which nanosized particles cause adverse health effects is their ability to generate ROS, which leads to oxidative stress in cells [10, 11]. As shown in Fig. 4, the relative frequencies of ROS positive cells to the solvent control increased in a dose-dependent manner in all cells treated for 1 h with kaolin particles. After treatment with 200 μg/mL kaolin micro-particles, the fluorescence frequencies in CHO AA8, HEKn and FJ cells rose to 1.3, 1.2 and 1.2, respectively. Kaolin nano-particle-treatment (200 μg/mL) of CHO AA8, HEKn and FJ cells increased the fluorescence frequencies to 1.5 1.2 and 1.3, respectively. Two-way ANOVA revealed significant main effects of concentration and particle size on ROS production (both *p* < 0.05) in all cells, and their interaction effect was also significant (*p* < 0.05) in all cells. We observed that kaolin nano-particles induced more ROS than micro-particles in all cells. Governa et al. reported high ROS generations in human polymorphonuclear leucocytes and bovine alveolar macrophagues treated with kaolin [12]. The mechanism of ROS production by kaolin particles is still unclear. In part, a numerous active sites on the large surface area might capture oxygen molecules and produce ROS through dismutation or Fenton reaction with impurity Fe as proposed in silica nanoparticles [13]. Furthermore, Suzuki et al. reported that fine metal particles show a higher cellular uptake than coarse particles [14]. Although we did not evaluate the uptake of kaolin particles, the ROS induction level might depend on the amount of incorporation. What is more, we previously showed that addition of the anti-oxidative agent *N*-acetyl cysteine reduced the frequency of MN induced by kaolin nanoparticles in CHO AA8 cells, indicating that oxidative stress is involved in genotoxicity [4]. Taken together, ROS induced by kaolin particles may also cause DNA damage resulting in MN formation in primary diploid dermal cells.

## Conclusion

Kaolin particles dose-dependently induced MN, DNA damage and ROS in primary normal human epidermal keratinocytes, dermal fibroblasts and Chinese hamster ovary cell line cells, with no significant differences in induction of toxicity. Fine particles of kaolin demonstrated higher genotoxic potency than coarse particles. Studies on the transdermal genotoxicity of kaolin particles are scarcer than those on its genotoxic effects toward respiratory organs/cells [15–17], and to the best of our knowledge this is the first report of in vitro genotoxicity analysis using human primary diploid dermal cells. The findings of the present study will contribute to safety evaluation of kaolin as a white cosmetic pigment.

## Data Availability

Not applicable.

## Abbreviations

ANOVA: Analysis of variance
CHO: Chinese hamster ovary
MMC: Mitomycin C
MN: Micronucleus
PBS: Phosphate buffered saline
ROS: Reactive oxygen species
